# Editorial on the Special Issue “Harmful Algal Blooms (HABs) and Public Health: Progress and Current Challenges”

**DOI:** 10.3390/toxins7114437

**Published:** 2015-10-30

**Authors:** Lesley V. D'Anglada

**Affiliations:** U.S. Environmental Protection Agency, Office of Science and Technology, Office of Water, 1200 Pennsylvania Ave., N.W., Washington, DC 20460, USA; E-Mail: Danglada.lesley@Epa.gov

Harmful Algal Blooms (HABs) affect the quality of fresh and marine waters and adversely affect both animals and humans. Public health risks include exposure to toxins through consumption of contaminated drinking water and fish and shellfish, and by recreating on or in contaminated waters. Federal and State professionals and researchers contributed to this Special Issue on HABs and Public Health with research papers and reviews on various aspects of public health including the occurrence and fate of toxins in the environment, monitoring efforts in freshwater and marine water systems, human health risk assessment, effectiveness of treatment techniques, and guideline development. 

Understanding the processes cyanobacteria and their toxins undergo in the environment is considered in the papers by Schmidt *et al.* [1], Fadel *et al.* [2], and Song *et al.* [3]. Schmidt *et al.* [1] discussed the environmental fate of microcystins, cyanobacterial toxins, and their toxicokinetics (absorption, metabolism, distribution, and excretion) in the body. Regarding environmental fate, the authors not only discussed the process of photodegradation of microcystins, but also the contribution of bacterial degradation that transforms the parent compounds into a series of conjugated products. This detoxification process, which according to the authors, is not well understood, could form toxic conjugates. Toxin degradation is also considered in the paper by Fadel *et al.* [2]. The authors recorded the degradation of cylindrospermopsin, another cyanobacterial toxin, due by sedimentation in lakes or by degradation, even in the presence of cylindrospermopsin-forming cyanobacterium blooms. This research emphasized that although it was not possible to definitely determine the relationship of cylindrospermopsin with other environmental factors, such as nutrients, water levels and temperature, the toxin was not correlated with cyanobacterium biovolume since it was observed at high concentrations even long after the cyanobacterium bloom had senesced. Sedimentation of microcystin in lakes, and their relationship with biological and physicochemical variables was explored by Song *et al.* [3]. Microcystin was detected in all sediment samples, and spatial variability was observed among microcystins and cyanobacterial biomass in different water levels and in sediments, highlighting the importance of the interaction between water and sediments in the distribution of microcystins in aquatic systems.

HABs have an adverse impact in recreational waters by fouling beaches and shoreline, affecting the quality of the water, and limiting recreational activities such as fishing, swimming, and boating. The adverse effect of the occurrence of both marine and freshwater toxic algal blooms in recreational waters in Washington State was discussed by Trainer and Hardy [4], with a focus on monitoring efforts and the role of these efforts in the protection of public health. In addition to regular monitoring practices for cyanotoxins, the authors described the effectiveness of partnering state regulatory programs with citizen and user-fee sponsored monitoring efforts in the surveillance and reporting of HABs and how the combination of technologies provides a comprehensive system for the protection of public health from exposure to HABs in fresh and marine waters. Trainer and Hardy also discussed the role of forecasting systems for marine and freshwater HABs, the basis for Wynne and Stumpf’s paper [5]. Wynne and Stumpf discussed in their paper the usefulness of satellite data to examine spatial patterns of blooms and help local communities and managers in planning. Satellites may help managers identify patterns of bloom development and the areas most commonly impacted, some of them being public water supplies or recreational areas. Spatial and temporal distribution of blooms is also the topic of the paper by Van de Merwe and Price [6], though here the emphasis is on the use of data from unmanned aircraft systems, then to correlate it with cyanobacterial biomass densities at the water surface. The authors demonstrate how these methods can provide valuable information that could help improve risk assessments and risk management derived from traditional risk assessment methods.

HABs also could be present in drinking water and could potentially affect drinking water treatment. Taste-and-odor problems have led some utilities to change processes during the drinking-water treatment to decrease tastes and odors in finished drinking water caused by algal blooms in the supply reservoir. Another problem is the presence of cyanobacterial cells and toxins in finished drinking water. In the paper by Szlag *et al.* [7], the authors concluded that conventional treatment effectively removed cyanobacterial cells and toxins. The authors conducted monitoring of three toxins (microcystins, anatoxin-a, and cylindrospermopsin), and toxin-producing cyanobacteria on raw and finished water samples from five conventional drinking water treatment plants experiencing cyanobacterial blooms in their raw water. One of the toxins, anatoxin-a, was not detected in any of the utilities, and all finished water samples showed toxins levels below the analytical methods detection limits. 

Human health risks from exposure to HABs is another topic discussed in this special issue. The paper by Hilborn and Beasley [8] used harmful cyanobacteria-associated animal illnesses and deaths as sentinel events to warn of potential human health risks. The paper primarily focuses on the One Health concept to integrate and collaborate among disciplines as a way to effectively monitor environmental and animal health as a way to assess human health risks. The authors concluded that illnesses or deaths among livestock, dogs, and fish are all potentially useful as predictors for the presence of cyanobacteria-associated human health risks. Human health risks surveillance is also the topic of the paper by Backer *et al.* [9], with a focus on the reports from States describing bloom events and associated adverse human and animal health events collected in the Harmful Algal Bloom-related Illness Surveillance System (HABISS) from 2007 to 2010. States used monitoring data to develop a wide range of public health prevention and response activities including issuing public health advisories or beach closures, and the development of public outreach activities. This work is indicative of the need of attention to public health risks associated with human and animal exposures to cyanobacteria and algae blooms. As mentioned before, HABs can cause adverse health effects in both humans and animals as recorded in Kansas by Trevino-Garrison *et al.* [10]. In this paper, the authors described the human and animal HAB-associated health events in 2011, including reports of dog illnesses and several deaths, and human illnesses, some of them requiring hospitalization. As part of its surveillance activities, the Kansas Department of Health and Environment, in conjunction with their local and national partners, developed a Harmful Algal Bloom Policy and Response Plan. This plan included the investigation of reports of HAB-related cases, the evaluation of water sample data, and education to the public of the public health risks. The authors highlighted the importance of the development of policies and guidelines to prevent morbidity and mortality among humans and animals. 

Numerous techniques already exist for managing blooms in reservoirs. However, the effectiveness of these techniques is relative. For example, Bauza *et al.* [11] exposed water samples from a recreational lake with cyanobacteria to different concentrations of hydrogen peroxide. Densities of cyanobacterial cells collapsed after exposure to the highest concentration over a 48 hour period in the presence of light. The authors concluded that the use of hydrogen peroxide could be used in hypertrophic systems. As in Bauza *et al.* [11], the paper by Lürling *et al.* [12] also evaluates the effectiveness of hydrogen peroxide to reduce cyanobacterial cells and their toxins in freshwater systems, albeit this time also evaluating the effectiveness of ultrasound. Peroxide effectively reduced toxin-producing cyanobacteria biomass at similar levels to those found by Bauza *et al.*, and proved to be ineffective at low levels. However, although a reduction of toxins was observed, still a significant release of the toxins into the water was detected. Ultrasound treatment only caused minimal growth inhibition and some release of toxins into the water, showing the treatment to be ineffective at controlling cyanobacteria. In these proposals, toxin reducing bacterial strains are used in water reservoirs as another option that may help in the reduction of microcystins occurrence. The use of bioreactors to eliminate microcystins is suggested by Dziga *et al.* [13] as an alternative to chemical methods of cyanotoxins elimination. This paper describes the effectiveness of using genetically engineered bacteria to degrade microcystins, based on further research on the optimization of the technique and to follow-up the long-term stability of the designed systems in natural conditions. 

This special issue also includes a paper describing the development of guideline values for cyanotoxins in the state of Oregon. In the United States, drinking water contaminants are regulated under the Safe Drinking Water Act (SDWA). Currently, there are no regulations for cyanotoxins in drinking water under the SDWA, but EPA developed in June 2015, Health Advisories (HAs) for the cyanotoxins microcystins and cylindrospermopsin, to assist federal, state and local officials in protecting public health from exposure to these two toxins in drinking water systems. Regulations or guidelines have not been developed either for aquatic life, aesthetics, or recreation in any body of water under the Clean Water Act (CWA). In the absence of these guidelines, many US States, including Oregon (Ferrer *et al.* [14]) have developed guidelines for cyanotoxins. The Oregon Health Authority (OHA) developed guideline values for drinking water, human recreational exposure, and dog recreational exposures for the four most common cyanotoxins in Oregon’s fresh waters. This study shows that having cyanotoxin guidelines can give rise to the development of toxin-based monitoring programs, which reduce the number of health advisories, an important step in the protection of public health.

Public health professionals have taken measures to protect public health by assessing and monitoring HABs occurrence and health effects, developing guidelines and HAB-related public health programs, and implementing remediation and treatment technologies. Despite these efforts, it is reasonable to say that the factors that promote HABs and their toxin production, the health impacts, and the fate of these blooms and their toxins in the environment is not totally understood. The different studies published in this special issue recognized these knowledge gaps including the spatial variability among cyanobacteria and their toxins in water and sediments, the complexity of monitoring and inconsistency in treatment techniques, and the importance of the development of guidelines for the protection of public health. 
